# Preparation and Biodistribution Studies of a Radiogallium-Acetylacetonate Bis (Thiosemicarbazone) Complex in Tumor-Bearing Rodents

**Published:** 2012

**Authors:** Amir Reza Jalilian, Hassan Yousefnia, Kamaleddin Shafaii, Aytak Novinrouz, Amir Abbas Rajamand

**Affiliations:** *Agricultural, Medical and Industrial Research School (AMIRS), Nuclear Science and Technology Research Institute Karaj, P. O. Box 31485-498, Iran. *

**Keywords:** Gallium-67, Acetyl acetone bis- thiosemicarbazonate, Biodistribution, Fibrosarcoma

## Abstract

Various radiometal complexes have been developed for tumor imaging, especially Ga-68 tracer. In the present study, the development of a radiogallium bis-thiosemicarbazone complex has been reported. [^67^Ga] acetylacetonate *bis*(thiosemicarbazone) complex ([^67^Ga] AATS) was prepared starting [^67^Ga]Gallium acetate and freshly prepared acetylacetonate *bis *(thiosemicarbazone) (AATS) in 30 min at 90°C. The partition co-efficient and the stability of the tracer were determined in final solution (25°C) and the presence of human serum (37°C) up to 24 h. The biodistribution of the labeled compound in wild-type and fibrosarcoma-bearing rodents were determined up to 72 h. The radiolabled Ga complex was prepared in high radiochemical purity (> 97%, HPLC) followed by initial biodistribution data with the significant tumor accumulation of the tracer in 2 h which is far higher than free Ga-67 cation while the compound wash-out is significantly faster. Above-mentioned pharmacokinetic properties suggest an interesting radiogallium complex while prepared by the PET Ga radioisotope, ^68^Ga, in accordance with the physical half life, for use in fibrosarcoma tumors, and possibly other malignancies.

## Introduction

The interesting physical properties and availability of gallium-67 make it an interesting nuclide for radiopharmaceutical research (1). The increasing trend in the production and use of PET gallium nuclides in nuclear medicine has offered new opportunities for researchers to focus on the production of new ^67^Ga-radiopharmaceuticals for feasibility studies for their future PET gallium homologs.

The auger electrons emitted by ^67^Ga possess potent cytotoxicity pointing towards potential therapeutic applications of the radionuclide (2), while the positrons emitted by ^68^Ga may also have therapeutic applications in the prevention of restenosis by intra-coronary radiation therapy (3).

Thiosemicarbazone gallium complexes have shown interesting anti-proliferative activity *in-vitro* and *in-vivo* (4). The most studied compounds are nitrogen-containing heterocycles (5), which is possibly due to their resemblance to pyridoxal metabolites that attach to co-enzyme B_6_-dependant enzymes and cause enzyme inhibition (6). Various gallium-based radiotracers have been reported by Green *et al*. (7-9), including an acetoacetate gallium-67 complex as a potential radiopharmaceutical (10).

However, there are rare examples of gallium bisthiosemicarbazones according to our knowledge, while copper analogs of bisthiosemicarbazones have been extensively studied (11, 12). Their biological activity and structure-activity have been well reported and the retention mechanisms in hypoxic and normoxic tumors are cited.

Usually, traditional bisthiosemicarbazones such as ATSM, PTSM, *etc.*, do not form complexes with gallium due to many chemical and molecular orbital considerations. However in our experiments, we were able to detect interesting radiolabeling properties for acetylacetoacetate bisthiosemicarbazone homologs. Such complexes were not reported by others while the reports on the copper and nickel complexes are lately reported (13).

Recently, we have reported the radiosynthesis of ^67^Ga-labeled pyridine-based thiosemicarbazone (^67^Ga-APTSM_2_) (14) as well as its evaluation in the fibrosarcoma bearing mice using scarification and SPECT studies (15).

**Figure 1:**
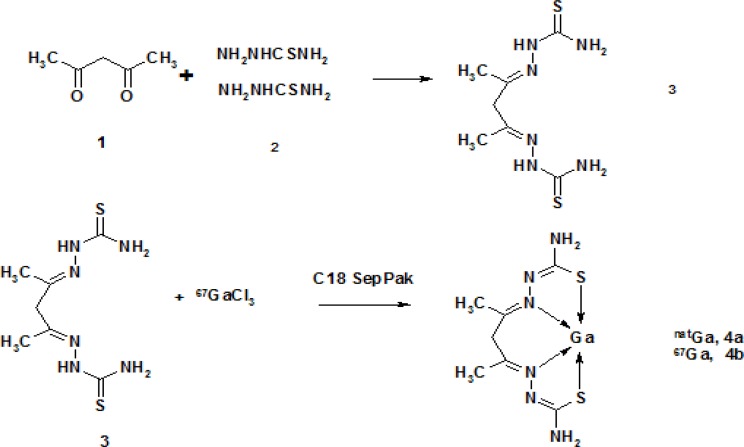
Synthesis of [^67^Ga] AATS

Due to the interesting anti-neoplastic activity of gallium thiosemicarbazones and the possibility of developing new series of ^67^Ga-labeled acetylacetate bis-thiosemicarbazonate complexes as possible tumor imaging agents using SPECT, the production, purification and biodistribution studies of [^67^Ga]-AAPS were investigated (Figure 1).

## Experimental

Enriched zinc-68 chloride with a purity of more than 95% was obtained from Ion Beam Separation Group at Agricultural, Medical and Industrial Research School (AMIRS). Production of ^67^Ga was performed at the Nuclear Medicine Research Group (AMIRS) 30 MeV cyclotron (Cyclone-30, IBA). Other chemicals were purchased from the Aldrich Chemical Co. (Aldrich, Germany) and the ion-exchange resins were purchased from Bio-Rad Laboratories (Canada) Ltd. NMR spectra were obtained on a FT-80 Varian instrument (80MHz) with tetramethylsilane as the internal standard. Infrared spectrum was measured on a Perkin-Elmer 781 spectrometer using a KBr disc. Mass spectrum was recorded by a Finnigan Mat TSQ-70 Spectrometer. Thin layer chromatography (TLC) for cold compounds was performed on polymer-backed silica gel (F 1500/LS 254, 20×20 cm, TLC Ready Foil, (Schleicher and Schuell , Germany)). Normal saline and sodium acetate used for labeling were of high purity and had been filtered through 0.22 µm Cativex filters. Instant thin layer chromatography (ITLC) was performed by counting Whatman No. 2 papers using a thin layer chromatography scanner, Bioscan AR2000, Bioscan Europe Ltd. (Bioscan, France). Analytical high performance liquid chromatography (HPLC) used to determine the specific activity, was performed by a Shimadzu LC-10AT, armed with two detector systems, flow scintillation analyzer (Packard-150 TR) and UV-visible (Shimadzu) using Whatman PartiSphere C-18 column 250 × 4.6 mm, Whatman, NJ (USA). Biodistribution data were acquired by counting normal saline washed tissues after weighting on a Canberra™ high purity germanium (HPGe) detector (model GC1020-7500SL). Radionuclidic purity was checked with the same detector. For activity measurement of samples, a CRC Capintech Radiometer (NJ, USA) was used. All calculations and ITLC counting were based on the 184 keV peak. Animal studies were performed in accordance with the United Kingdom Biological Council›s Guidelines on the Use of Living Animals in Scientific Investigations, 2^nd^ Edn.

*Production of *^67^*Ga*

^68^Zn (p, 2n) ^67^Ga was used as the best nuclear reaction for the production of ^67^Ga. Other impurities could be removed in the radiochemical separation process. After the target bombardment process, chemical separation was carried out in no-carrier-added form. The irradiated target was dissolved in 10 M HCl (15 mL) and the solution was passed through a cation exchange resin (AG 50W, H^+^ form, mesh 200-400, h:10 cm, Ø:1.3 cm) which had been preconditioned by passing 25 mL of 9 M HCl.

The column was then washed by 25 mL of 9 M HCl at a rate of 1 mL/min to remove the copper and zinc ions. Thirty mL water plus about 100 mL of a 6 M HCl solution was added to the eluent. The latter solution was loaded on another exchange resin (AG1X8 Cl- form, 100-200 mesh, h: 25 cm, Ø: 1.7 cm) pretreated with 6 M HCl (100 mL). Finally, the gallium-67 was eluted as [^67^Ga] GaCl_3_ using 2 M HCl (50 mL). The whole process took about 60 min.

*Quality control of the product*

*Control of radionuclide purity*

Gamma spectroscopy of the final sample was carried out counting in an HPGe detector coupled to a Canberra™ multi-channel analyzer for 1000 sec.

*Chemical purity control*

The presence of zinc cation was detected by visible colorimetric assays. Even at 1 ppm of standard zinc concentration, the pinkish complex was visible to the naked eye, while the test sample remained similar to the blank (16). The amount of copper cation was checked in the final solution using color formation with dithizone reagent (17).

*Production of acetylacetonate bis (thiosemicarbazone) (AATS) (* 3 *)*

This compound was prepared with slight modifications to the reported method (18). Briefly, to a transparent stirring mixture of thiosemicarbazide (2 mmol) (2) in 5% acetic acid at 50°C, freshly distilled acetylacetone (1 mmol) (1) was added drop-wise for 5 min. The mixture was stirred for another 30 min at 50°C. The reaction mixture was cooled down in an ice bath and the precipitate was filtered. The precipitate was washed with water (10 mL) and ethanol (20 mL) and finally dried in oven at 70-80°C for at least 8 h. The residue can be further purified by refluxing the mixture of the precipitate in 80% acetic acid at 50-70°C for 10-14 h. The filtered mass was heated in an oven at 80°C and finally crystallized from hot ethanol to give a light yellow powder (60%) m.p. 148-150°C. ^1^H NMR (CDCl_3_)   (ppm) 9.19 (s, 1H, NH), 7.91 (s, 2H, -CH_2_-), 2.86 (d, 6H, CH_3_-C=N). IR (CHCl_3_)  max 3535, 3129, 2956, 2495, 2361, 1563, 1399, 1215, 1041, 661, 556. Mass (electrospray) 190.1 (M^+^).

*Preparation of [* ^67^ *Ga] acetylacetonate bis(thiosemicarbazone) complex ([* ^67^ *Ga]AATS)*

The acidic solution (2 mL) of [^67^Ga]GaCl_3_ (111 MBq, 3 mCi) was transferred to a 5 mL-borosilicate vial containing 0.5 mL of acetate buffer (pH = 5.5). Fifty µL of acetylacetonate bis(thiosemicarbazone) (AATS) in absolute ethanol (1 mg/mL 260 nmoles) was added to the gallium-containing vial and vortexed at 80-90°C for 30 min. The mixture was then cooled to room temperature. The vial mixture was diluted by the addition of normal saline (4.5 mL). The active solution was checked for radiochemical purity by ITLC and HPLC. In case of high free gallium content presence, the mixture (about 5 mL) was cooled in an ice bath and rapidly injected into a C_18_ Sep-Pak column pretreated with 5 mL of ethanol and 2 mL of water. The column was washed with water (4 mL) and purged with a stream of dry N_2_. The labeled compound was finally eluted using 0.2 mL-portions of absolute ethanol and the fractions were counted in HPGe detector. The vial containing the maximum radioactivity was diluted to a 5% solution by addition of normal saline followed by passing through a 0.22 µm filter and pH was adjusted to 5.5-7.

*Quality control of [* ^67^ *Ga] AATS*

*Radio thin layer chromatograph*

A 5 µL sample of the final fraction was spotted on a chromatography Whatman No. 2 paper and developed in 10% ammonium acetate: methanol (1 : 1) mixture as the mobile phase.

*High performance liquid chromatography*

HPLC was performed with a flow rate of 1 mL/min (pressure = 130 KgF/cm^2^ for 20 min). Radiolabeled compound was eluted using a mixture of two solutions (A: acetonitrile + 0.1% TFA/water + 0.1% TFA, 90 : 10) using reversed phase column Whatman PartiSphere C_18_ 4.6 × 250 mm.

*Stability of [* ^67^ *Ga]AATS complex in the final product*

Stability tests were based on previous studies performed for radiolabeled metal complexes (19). A sample of [^67^Ga] AATS (5 mCi) was kept at room temperature for 4 days while checked by RTLC every half an hour. A micropipette sample (5 µL) was taken from the shaking mixture and the ratio of free radiogallium to [^67^Ga]AATS was checked by instant thin layer chromatography.

*Serum stability studies*

A mass of 36.1 MBq (976 Ci) of [^67^Ga]AATS was added to 500 µL of freshly prepared human serum and the resulting mixture was incubated at 37°C for 2 days. Aliquots (5µL) were analyzed by ITLC after 0, 0.25, 0.5, 1, 2 and 3 h of incubation to determine the stability of the complex.

*Determination of partition coefficient*

The partition coefficient of the [^67^Ga]AATS was measured following 1 min of vigorous vortex mixing of 1 mL of 2-octanol and 1 mL of isotonic acetate-buffered saline (pH = 7) with approximately 3.7 MBq (100 µCi) of the radiolabeled copper complex at 37°C. Following further incubation for 5 min, the octanol and aqueous phases were sampled and counted in an automatic well counter. A 500 µL sample of the octanol phase from this partitioning was repartitioned 2-3 times with fresh buffer to ensure that the traces of hydrophilic ^67^Ga impurities did not alter the calculated p- values.

The reported log p- values are the average of the second and third extractions from three to four independent measurements. Log p-values represent the mean (standard deviation) of five measurements.

*Induction of fibrosarcoma tumors in rodents*

Tumor induction was performed by the use of poly aromatic hydrocarbon injection in rodents as reported previously (20).

For tumor model preparation, 10 µL of 3-methylcholanthrene solution in extra-virgin olive oil (4 mg/mL) was injected SC to the dorsal area of the mice. After 14-16 weeks, the tumor weighed 0.2-0.4 g and was not grossly necrotic. Tumor tissues of some random animals were sent for pathological tests and were diagnosed as fibrosarcoma.

*Biodistribution in wild-type and fibrosarcoma-bearing animal tissues*

The distribution of the radiolabeled complex among tissues was determined for normal rats. The total amount of radioactivity (35 ± 2 *µ*Ci) injected into each rat was measured by counting the 1 mL syringe before and after the injection in a dose calibrator with fixed geometry.

The animals were sacrificed by CO_2_ asphyxiation at selected times after the injection (0.5, 1, 2, 24 and 48 h). The tissues (blood, heart, lung, brain, intestine, feces, skin, stomach, kidneys, liver, muscle and bone) were weighed and rinsed with normal saline and their specific activities were determined with an HPGe detector equipped with a sample holder device as the percentage of injected dose per gram of tissues.

**Figure 2:**
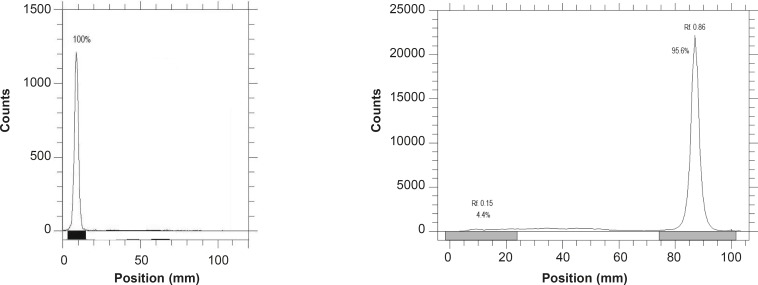
ITLC of [^67^Ga] GaCl_3_ (left) and [^67^Ga]AATS (right) on Whatman No. 2 paper developed in 10% ammonium acetate : methanol (1 : 1).

## Results and Discussion

Although there are reports on the reaction of acetylacetone and thiosemicarbazides in the literature showing that the reaction is complicated by the possible formation of pyrazoline forms (21), it has fortunately been shown that these compounds in the presence of mild acidic conditions (pH = 4-6) and metallic ions, afford the metallic bis-thiosemicarbazonate complexes (22). These studies are mostly performed in presence of copper and nickel cations; however, it is very possible that a similarity would be observed for Ga cation as well. A major drawback in copper complexes has shown to be the oxidation of the methylene backbone of the complex while in 15 h, the oxidized species can be observed (17) leading to the formation of Cu^+^ cation. We did not have any proofs regarding the oxidation of the Ga complexes; however, the whole radiolabeling procedure was performed under N_2_ atmosphere to avoid possible oxidation. According to our knowledge, there are no reports of other Ga oxidation states in the literature.

*Production*

Gallium-67, as GaCl_3_, was prepared by 24 MeV proton bombardment of the ^68^Zn target at Cyclone-30 on a regular basis. The target was bombarded with a current intensity of 170 µA and a charge of 1400 µAh. The chemical separation process was based on a no-carrier-added method.

Radiochemical separation was performed by a two-step ion exchange chromatography method with a yield of higher than 95%. Quality control of the product was performed in two steps. Radionuclidic control showed the presence of 93(40%), 184(24%), 296(22%) and 378(7%) keV gamma energies, all originating from ^67^Ga and showed a radionuclidic purity higher than 99% (E.O.S.). The concentrations of zinc (from target material) and copper (from target support) were determined using visible colorimetric assays and shown to be below the internationally accepted levels, *i.e.* 1 ppm Zn (14) and 5 ppm for Cu (15).

*Radiolabeling of [* ^67^ *Ga] AATS*

Labeling the AATS with a gallium cation, affects its chromatographic properties because of the engagement of several polar functional groups in its structure and the final complex is also more lipophilic. Therefore, free gallium remains at the origin (Rf. 0.0), while the radiolabeled complex migrates to a higher Rf (0.86) (Figure 2).

**Figure 3:**
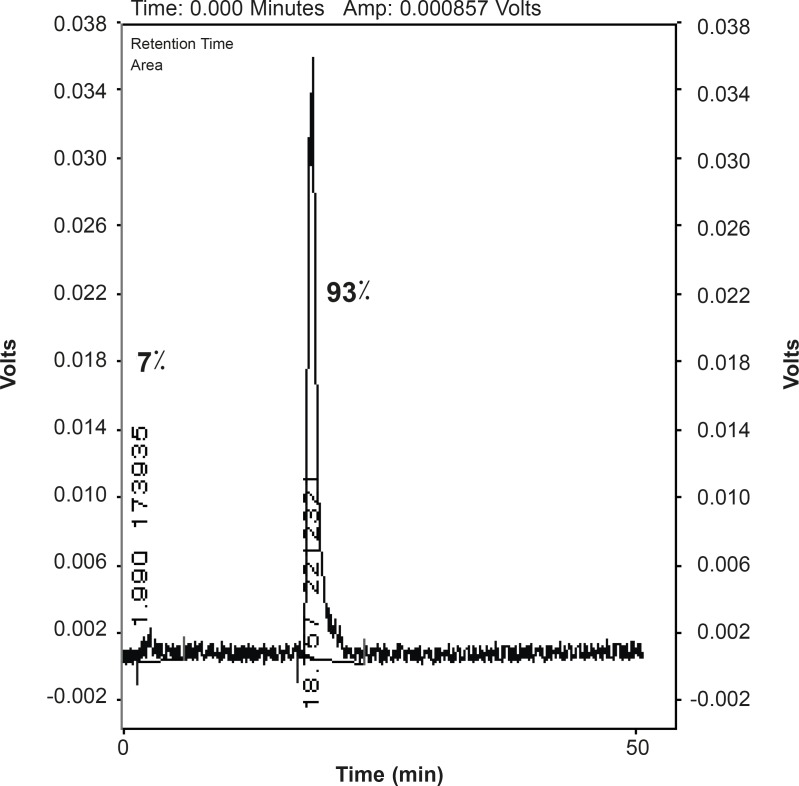
HPLC chromatogram of [^67^Ga] AATS solution on a reversed phase column using acetonitrile + 0.1%TFA/water + 0.1% TFA, 90:10.

The lipophilic nature of the complex was a major reason in HPLC distinct radioanalysis and a reverse phase column was preferable. Free Ga was eluted at 1.99 min while the complex was eluted at 18.57 min demonstrating a radiochemical purity of 93% using optimized conditions without further purifications (Figure 3).

**Figure 4:**
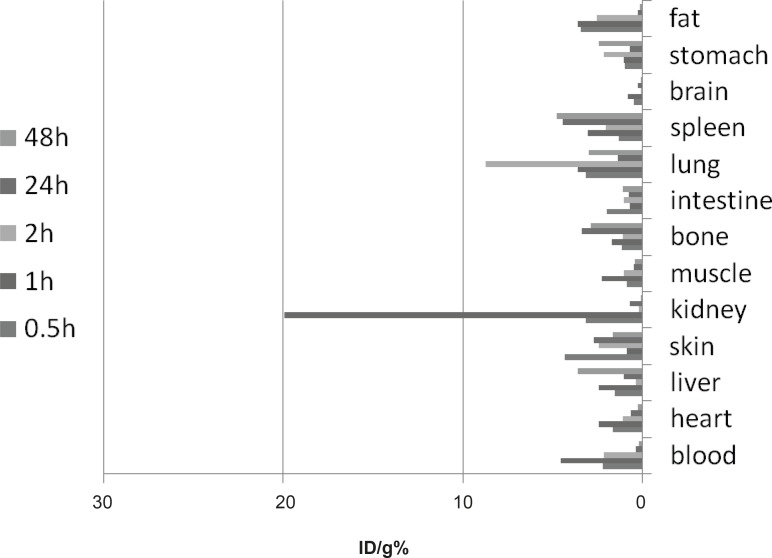
Biodistribution of [^67^Ga] AAPS (1.85 MBq, 35±2 µCi) in wild-type mice (n = 3, SD: ± 2%) 0.5-48 h after IV injection via tail vein (ID/g%: percentage of injected per gram calculated using area under curve of 184 keV peak in gamma spectrum).

**Figure 5:**
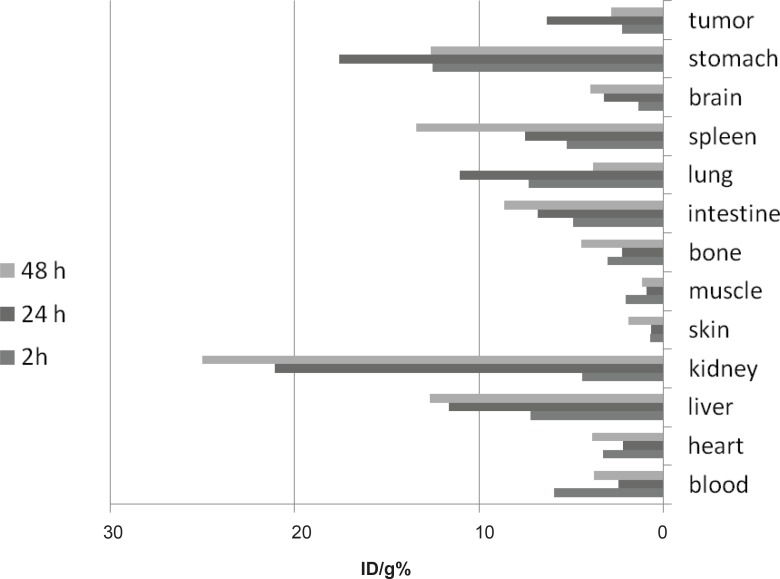
Biodistribution of [^67^Ga]AAPS (1.85 MBq, 38µCi) in fibrosarcoma-bearing mice (n = 3, SD: ± 2%) 248- h after IV injection via tail vein ID/g%: percentage of injected per gram calculated using area under curve of 184 keV peak in gamma spectrum).

*Optimization*

No detectable complex was formed at room temperature. The best temperature was found to be 85-90°C. At this temperature, when freshly prepared gallium-67 was used, all the radio-gallium was inserted into the complex. While heating the reaction mixture over 100°C or for more than 1 h, the radiochemical yield dropped. The final radiolabeled complex in alcoholic media was diluted in normal saline to a 5% solution.

The solution was stable at room temperature up to 4 days post-formulation, allowing performance of biological experiments. Before the experiments, the solution passed through a 0.22 microns filter (Millipore).

*Stability*

The chemical stability of [^67^Ga]AATS was high enough to perform further studies. Incubation of [^67^Ga] AATS in freshly prepared human serum for 2 days at 37°C showed no loss of ^67^Ga from the complex. The radiochemical purity of complex remained at 95 ± 3% for 4 days under physiologic conditions.

*Biodistribution*

One hour post-injection, the radioactivity enhanced in the kidneys (Figure 4). This pattern rapidly drops after 2 h. The radioactivity of intestine, as well as GI tract, was high at 2 h which could be due to the metabolism of the radiolabeled complex in the liver. The pattern for sternum, skin and brain remained almost unchanged. The major excretion route for the tracer was urinary tract as shown in Figure 4. A clear accumulation occurred in the spleen and the reticuloendothelial system after 24 h possibly due to the biodistribution of free Ga^3+^.

The biodistribution data for radiolabeled compound in fibrosarcoma-bearing mice is presented in Figure 5. The tumor uptake was not significant at least 1 h post-injection; however, a major activity content (6-7%) was accumulated in the tumor mass after 2 h. But after 48 h, the tumor uptake fades. This is an interesting result since compared to the Ga^3+^ cation, the tumor uptake usually increases after 24-48 h due to various mechanisms suggested such as transferrin receptors and/or acidity of the tumor cells (23).

In case of gallium being transferred through transferrin route, it would take at least 24 to 48 h; therefore, the tumor accumulation is not being mediated by free Ga cation released from complex and/or other sources after metabolism. This accumulation is taking place as another route that must be studied further.

## Conclusions

Total labeling and formulation of [^67^Ga]AATS took about 40 min, with a radiochemical purity of higher than 93% (HPLC). A significant specific activity (9.1 TBq/mmol or 246 Ci/mmol) was formed via insertion of ^67^Ga cations. The radiolabeled complex was stable in aqueous solutions for at least 4 days and 2 days in presence of human serum and no significant amount of other radioactive species was detected by ITLC, 12 h after labeling. Trace amounts of [^67^Ga]gallium cation (4%) were detected by ITLC indicating that radiochemical purity of the [^67^Ga]AATS was higher than 96%. In HPLC studies, a radiochemical purity of 93% was detected. The biodistribution of the tracer in wild-type rats demonstrated that the major route of excretion is a urinary tract. The tracer afforded significant tumor uptake (7%) after 24 h in fibrosarcoma-bearing mice. [^67^Ga]AATS can be a potential SPECT radiotracer for malignancy imaging. Further investigations on other tumor models and trapping mechanisms are required while production of ^68^Ga homolog can be of great interest for PET studies.
